# Mortality patterns in Southern Adriatic islands of Croatia: a registry-based study

**DOI:** 10.3325/cmj.2018.59.118

**Published:** 2018-06

**Authors:** Joshua Rehberg, Ana Stipčić, Tanja Ćorić, Ivana Kolčić, Ozren Polašek

**Affiliations:** 1 Medical student, Medical School, Medical College of Wisconsin, Milwaukee, WI, USA; 2 Department of Health Studies, University of Split, Split, Croatia; 3 Croatian National Institute of Public Health, Zagreb, Croatia; 4 Department of Public Health, University of Split School of Medicine, Split, Croatia; * The first two authors contributed equally.

## Abstract

**Aim:**

To investigate the mortality patterns on the Southern Adriatic islands of Croatia and compare them with those in two, mainly coastal, mainland counties.

**Methods:**

In this registry-based study we used the official mortality register data to analyze the mortality patterns on seven Croatian islands (Brač, Hvar, Korčula, Lastovo, Mljet, Šolta, and Vis) and Pelješac peninsula in the 1998-2013 period and calculated the average lifespan, life expectancy, and standardized mortality ratios (SMR). We compared the leading causes of death with those in the mainland population of two southernmost Croatian counties.

**Results:**

The average lifespan of the island population was 3-10 years longer for men and 2-7 years longer for women than that on the mainland. All-cause SMRs were significantly lower for both men and women on Korčula, Brač, Mljet, and Pelješac but significantly higher for women on Šolta (1.22; 95% confidence intervals 1.07-1.38). The leading causes of death on the islands were cardiovascular diseases, with higher percentages in men and lower in women in comparison with those on the mainland. There were no substantial differences in the life expectancy at birth.

**Conclusions:**

Despite longer lifespan, lack of differences in life expectancy at birth suggests that the recent generations of islanders no longer show beneficial mortality patterns, possibly due to diminishing adherence to the Mediterranean diet and lifestyle. Restoring the traditional lifestyles is a public health priority, with the ultimate aim of reducing inequalities and improving the health of island inhabitants.

Mortality analysis is one of the critical tools in public and population health. Implications drawn from these analyses reflect a variety of events on a population scale and the performance and quality of health care. Furthermore, these analyses are of utmost importance in specific populations, including isolated and remote populations.

Croatia has a well-developed coastal profile, with over 1000 islands, many of which are inhabited. This is a unique and diverse setting for research (1-5), but it also causes a logistical and management problem for health care authorities, which in order to develop and implement effective health policies need to be aware of each population’s needs and health and disease profiles (6).

More specifically, the inhabitants of Southern Adriatic islands of Croatia present an intriguing population for analysis. Given their geographical isolation, founder effect and genetic drift have contributed to the development of several notable genetic profiles and disorders (5). In addition to their low genetic diversity, isolated populations such as these provide a valuable resource for health research given their low immigration rates and decreased environmental diversity (7-9). The geographical location also contributes to their uniqueness; they are the southernmost Croatian islands, located nearest to the Mediterranean Sea, with a history of typical Mediterranean diet consumption (10). Mediterranean population health has been the subject of numerous studies given the low mortality rates and high life expectancies at birth (11). A diet composed of a low saturated fat intake with a high monounsaturated fat intake, and a large amount of vegetables and fruits is the suggested factor contributing to the improved health of the Mediterranean population (12), as opposed to the most of mainland population (13).

Croatian islanders were found to have significantly higher mean age and lower mortality rates than the general or mainland Croatia populations (14). However, given the increasing disparity between the traditional Mediterranean diet and the current lifestyle trends on the Croatian islands (10,15-17), it remains to be seen whether the mortality rates remain lower than on the mainland. Such gradients have previously been described in many health-related outcomes (16,18-20), but to our knowledge there have been no targeted studies that could deliver useful information for policy making. Our aim was to investigate mortality profiles consisting of several analytical measures for seven Dalmatian islands and one peninsula and compare them with the mainland population of two southernmost, mainly coastal, counties of Croatia.

## Material and methods

We used mortality data from the Mortality Register maintained by the Croatian Institute of Public Health. The study encompassed all 1998-2013 death records of the residents of Split-Dalmatia County and Dubrovnik-Neretva County. We classified all death cases into nine groups, ie, seven groups for the selected islands (Brač, Hvar, Korčula, Lastovo, Mljet, Šolta, and Vis), one for the peninsula of Pelješac, and one for the mainland population, which consisted of the pooled data for the remaining population from the two counties. For the needs of this study, we treated Pelješac peninsula equally as islands since it is an equally isolated remote area. The two southernmost counties were used as the mainland controls, because previous studies suggested different health-related behaviors and outcomes in the coastal and continental Croatia (13,18,19).

To improve the study power, we pooled all the data across the entire 1998-2013 study period and divided it by the duration of the study to obtain average annual rates. This approach assumed lack of detectable time trends while allowing for the calculation of all estimates for all islands. Otherwise, most islands would have been excluded from the analysis due to small number or nonexistent deaths in certain age bands across the investigated years.

### Mortality indices

To assess the mortality patterns, we compared mortality from the leading causes of death, according to the ICD10 classification, for every island and the mainland counties. We also compared the average lifespans in the investigated populations, separately for men and women. We then calculated all-causes standardized mortality ratios (SMR) for every island to define the deviation of the island population from the populations of the two mainland counties. SMRs were calculated for each island vs mainland population (ie, excluding pairwise island comparisons). Lastly, we calculated the life expectancies at birth to provide unified account of the overall mortality profile for each island. Life expectancy was calculated for the entire 1998-2013 study period to increase power and reduce spurious results as a consequence of small sample sizes in certain age groups.

### Statistical analysis

Normality of variables’ distribution was tested by small-samples Shapiro-Wilk test. χ^2^ test was used for assessing the significance of differences between categorical variables and *t* test for numerical variables. The level of statistical significance was set at *P* < 0.05. All analyses were performed in IBM SPSS Statistics for Windows, version 19.0 (IBM Corp, Armonk, NY, USA licensed to the University of Edinburgh, UK).

## Results

Over the investigated period there were 91 954 death cases in the Split-Dalmatia and Dubrovnik-Neretva counties, 11 837 (12.9%) of which occurred in the investigated islands (Table 1). Mean age at death (average lifespan) of the island population strongly deviated from the mainland, with island men living 3-10 years longer and island women living 2-7 years longer than their mainland counterparts, with especially long lifespan observed on Mljet. The calculation of life expectancies across the investigated islands yielded rather unified results, without specific patterns. Mljet and Lastovo had somewhat higher estimates, but with a substantial overlap of confidence intervals, suggesting no difference from the mainland estimates (Table 1 and Supplementary Table 1)(supplementary Table 1).

SMR analysis suggested marked differences from the mainland, including significantly lower SMRs in both men and women on Korčula, Brač, Mljet, and Pelješac (Table 2). Significantly lower SMRs were found only in men on Lastovo and Hvar, while the remaining SMRs did not indicate any differences. A single exception was the island of Šolta, where women had significantly higher SMRs than the mainland population (Table 2).

**Table 2:** Standardized mortality ratios (SMR) based on all-cause mortality on the investigated Croatian islands and Pelješac peninsula in comparison with the mainland population

	SMR (95% confidence interval) in investigated regions							
Sex	Korčula	Vis	Lastovo	Hvar	Brač	Mljet	Šolta	Pelješac
Men	0.85 (0.81-0.89)	0.99 (0.91-1.08)	0.74 (0.60-0.91)	0.87 (0.82-0.92)	0.88 (0.83-0.92)	0.83 (0.71-0.98)	0.96 (0.84-1.08)	0.82 (0.77-0.88)
Women	0.93 (0.88-0.97)	1.05 (0.96-1.14)	0.96 (0.78-1.18)	0.98 (0.92-1.04)	0.90 (0.85-0.95)	0.69 (0.59-0.80)	1.22 (1.07-1.38)	0.89 (0.83-0.95)

The leading causes of death in men were cardiovascular diseases, with lower prevalence of tumors (Table 3). Overall, cardiovascular burden was higher on Korčula, Hvar, Brač, and Pelješac than on the mainland. There were no differences in tumor burden, with significantly lower burden of trauma on Brač. We recorded significantly lower burden of cardiovascular diseases in women on Brač and Šolta, significantly lower burden of tumors on Korčula, Hvar, and Pelješac, and significantly lower burden of trauma in women on Korčula (Table 3; *P* < 0.001 for all associations). The general pattern suggested higher cardiovascular burden in men, whereas the burden of all three main groups of death causes in women was lower in comparison with the mainland (Table 3).

**Table 3:** Proportional mortality for leading groups of causes of death in the investigated Croatian islands, Pelješac peninsula, and the mainland

	Proportional mortality in investigated regions (n,%)								
Causes of death	Korčula	Vis	Lastovo	Hvar	Brač	Mljet	Šolta	Pelješac	Mainland
Men									
cardiovascular	727 (47.0)	234 (47.0)	50 (58.0)	516 (46.0)	631 (46.0)	64 (42.0)	86 (35.0)	418 (50.0)	16 766 (41.0)
P*	<0.001	0.004	0.001	<0.001	<0.001	0.719	0.069	<0.001	-
tumors	466 (30)	142 (29.0)	19 (22.0)	320 (29.0)	435 (31.0)	47 (31.0)	85 (35.0)	226 (27.0)	12 891 (31.0)
P*	0.300	0.197	0.058	0.060	0.898	0.927	0.268	0.005	-
trauma and injury	95 (6.1)	23 (4.6)	5 (5.7)	65 (5.8)	61 (4.4)	10 (6.6)	12 (4.9)	53 (6.3)	2850 (6.9.0)
P*	0.227	0.046	0.668	0.152	<0.001	0.871	0.209	0.472	-
Women									
cardiovascular	943 (59.0)	301 (56.0)	58 (64.0)	654 (58.0)	722 (55.0)	118 (72.0)	113 (47.0)	571 (64.0)	23 898 (61.0)
P*	0.043	0.012	0.655	0.017	<0.001	0.003	<0.001	0.120	
tumors	344 (22.0)	109 (20.0)	15 (17.0)	200 (18.0)	278 (21.0)	23 (14.0)	51 (21.0)	146 (16.0)	9909 (26.0)
P*	<0.001	0.007	0.049	<0.001	0.001	0.001	0.125	<0.001	
trauma and injury	51 (3.2)	20 (3.7)	3 (3.3)	43 (3.8)	49 (3.8)	5 (3.1)	7 (2.9)	40 (4.5)	2000 (4.5)
P*	<0.001	0.141	0.426	0.045	0.025	0.237	0.116	0.378	

*χ^2^ test, island vs mainland.

## Discussion

Our results suggest a diverse mortality pattern on Southern Dalmatian islands. While the overall lifespan was longer on the islands with more favorable SMRs, we did not detect any differences in life expectancy at birth. When taken together, these results suggest that elderly islanders have been experiencing more favorable conditions, while in younger generations this protective “island” effect is reduced. These results agree with some previous studies, pointing out diminishing adherence to the Mediterranean diet and lifestyle (21-23). An alternative solution could be a “sick” migrant effect, which occurs when individuals with chronic diseases are moving to the coast, to be closer to the health care facilities. Opposed to this is the “healthy” migrant effect, occurring when healthy individuals take part in directional migration (24).

Disease-specific mortality rates also showed an interesting result, with higher cardiovascular diseases burden in men on the islands and correspondingly lesser burden for all three leading groups of death causes in women. One of the most parsimonious explanations for this could be their behavior, predominantly Mediterranean diet and lifestyle. This fits into the previous hypothesis of reduced health protection in recent generations, as several previous studies have described diminishing adherence to Mediterranean diet and worse medical outcomes (10,15,17). There could also be different underlying disease mechanisms due to genetic make-up of island populations. Having this in mind, the use of resources such as the 10 001 Dalmatians project could be a perfect example of translating basic research to provide opportunities for personalized medicine (5,25-27).

These results also confirm the previous finding of the island-specific disease profiles (5), which require the development and adherence to island-specific health policies. This is one of the most salient results of this study in terms of health care delivery and health policy, suggesting that “one size fits all” approach in terms of island health is insufficient, and that every island with larger permanent population should receive special attention and develop its own health strategy, in order to provide equitable health care across the entire population.

The first limitation of our study is that the mortality data analysis was encumbered by numerous effect modifiers, which can cause substantial deviations and must always be considered very carefully to prevent biased conclusions. The effect modifiers extend from the methodological limitations in the use of SMRs and life expectancies, which may have produced artifacts in the data analysis and interpretation (16). An additional possible cause of bias of unknown direction is the unknown extent of directional migrations. The second limitation stemmed from small sample sizes, leading to low statistical power, especially in the case of smaller islands. The observed mortality pattern might not reflect the morbidity patterns, especially if there are differences in clinical courses and outcomes between islands and the mainland. Lastly, there could be substantial differences in the quality of coroner services across the islands, possibly causing systematic bias toward higher cardiovascular burden. Nevertheless, this study suggests a transitional mortality pattern, marked by mostly better SMRs and comparable life expectancies, consistent with previously described lack of adherence to traditional Mediterranean diet and lifestyle. These results suggest that not all of the contemporary societal changes are beneficial, and that sometimes the old ways indeed are the best.

## 

**Table:** 

Mortality indices	Korčula	Vis	Lastovo	Hvar	Brač	Mljet	Šolta	Pelješac	Mainland
Number (%) of deaths	3152 (3.4)	1033 (1.1)	178 (0.2)	2247 (2.4)	2690 (2.9)	314 (0.3)	487 (0.5)	1736 (1.9)	80 117 (87.1)
Sex (n, %)									
men	1552 (49.2)	497 (48.1)	87 (48.9)	1118 (49.8)	1384 (51.4)	152 (48.4)	246 (50.5)	844 (48.6)	41 228 (51.5)
women	1600 (50.8)	536 (51.9)	91 (51.1)	1129 (50.2)	1306 (48.6)	162 (51.6)	241 (49.5)	892 (51.4)	38 889 (48.5)
*P*	0.014	0.032	0.491	0.111	0.992	0.280	0.677	0.019	-
Age at death (years; mean ± standard deviation)									
men	74.0 ± 26.5	74.2 ± 30.8	74.8 ± 13.4	73.0 ± 14.6	73.4 ± 14.4	79.1 ± 50.5	74.1 ± 12.4	74.0 ± 25.7	69.6 ± 17.9
P*	<0.001	<0.001	<0.001	0.009	<0.001	<0.001	<0.001	<0.001	-
women	80.8 ± 28.2	79.7 ± 11.8	81.9 ± 9.8	80.0 ± 12.3	79.1 ± 13.7	83.8 ± 10.4	80.7 ± 9.4	80.4 ± 12.1	77.1 ± 15.2
P*	<0.001	<0.001	<0.001	0.004	<0.001	<0.001	<0.001	<0.001	-
Life expectancy at birth (years; 95% confidence intervals)									
men	74.3 (71.3-77.4)	71.3 (64.4-78.1)	76.1 (65.4-86.7)	74.4 (71.0-77.7)	74.4 (71.3-77.4)	73.2 (60.2-86.1)	73.0 (64.0-82.0)	74.4 (70.1-78.7)	74.4 (73.9-74.9)
women	80.3 (77.8-82.8)	78.4 (72.7-84.1)	81.4 (73.8-89.0)	80.4 (77.4-83.3)	80.0(77.0-82.9)	83.2 (75.1-91.3)	79.7 (73.8-85.7)	80.8 (77.3-84.3)	80.9 (80.5-81.3)

*Pairwise comparisons of the island vs the mainland.
