# Supplementary material for: Mortality patterns in Southern Adriatic islands of Croatia: a registry-based study
Source: Croat Med J. 2018 Jun;59(3):118–23. doi: 10.3325/cmj.2018.59.118 (PMC6045898; doi:10.3325/cmj.2018.59.118)
Supplement: Supplementary Table 1 [file CroatMedJ_59_s001.pdf]

Life expectancies across age groups, with 95% confidence intervals

Men

Age group	Mainland	Korcula	Lastovo	Mljet	Hvar	Vis	Brač	Šolta	Pelješac
0-4	74.4 [73.9-74.9]	74.3 [71.3-77.4]	73.2 [60.2-86.1]	74.4 [71.0-77.7]	71.3 [64.4-78.1]	73.0 [64.0-82.0]	74.4 [70.1-78.7]	74.4 [71.3-77.4]	76.1 [65.4-86.7]
5-9	69.8 [69.3-70.2]	69.8 [67.1-72.6]	69.0 [57.6-80.4]	69.6 [66.4-72.8]	66.9 [60.8-72.9]	68.0 [59.0-77.0]	70.0 [66.1-73.9]	69.9 [67.3-72.5]	71.1 [60.4-81.7]
10-14	64.8 [64.4-65.3]	64.9 [62.2-67.7]	64.0 [52.6-75.4]	64.6 [61.5-67.8]	61.9 [55.8-67.9]	63.0 [54.0-72.0]	65.0 [61.1-68.9]	64.9 [62.3-67.5]	66.1 [55.4-76.7]
15-19	59.9 [59.4-60.3]	59.9 [57.2-62.7]	59.6 [49.3-70.0]	59.7 [56.6-62.8]	56.9 [50.8-62.9]	58.0 [49.0-67.0]	60.0 [56.1-63.9]	59.9 [57.3-62.5]	61.1 [50.4-71.7]
20-24	55.0 [54.6-55.5]	55.2 [52.5-57.8]	54.6 [44.3-65.0]	54.9 [51.9-58.0]	51.9 [45.8-57.9]	53.0 [44.0-62.0]	55.3 [51.6-59.0]	55.1 [52.6-57.7]	56.6 [46.7-66.5]
25-29	50.3 [49.8-50.7]	50.4 [47.9-53.0]	49.6 [39.3-60.0]	50.4 [47.5-53.2]	47.5 [41.8-53.2]	48.6 [40.8-56.4]	51.0 [47.6-54.3]	50.3 [47.8-52.8]	51.6 [41.7-61.5]
30-34	45.5 [45.1-45.9]	45.7 [43.2-48.2]	45.5 [36.1-54.9]	45.5 [42.8-48.3]	43.2 [37.9-48.5]	44.4 [38-50.9]	46.3 [43.0-49.5]	45.5 [43.1-47.9]	46.6 [36.7-56.5]
35-39	40.7 [40.3-41.1]	41.1 [38.7-43.4]	41.1 [32.8-49.4]	40.7 [38.1-43.4]	38.3 [33.1-43.5]	39.4 [33-45.9]	41.6 [38.5-44.7]	40.6 [38.3-43.0]	41.6 [31.7-51.5]
40-44	36.0 [35.6-36.4]	36.4 [34.2-38.7]	37.0 [29.7-44.4]	36.0 [33.4-38.6]	34.3 [29.6-38.9]	34.7 [28.6-40.8]	36.9 [34.0-39.8]	35.9 [33.6-38.2]	36.9 [27.4-46.5]
45-49	31.4 [31.0-31.8]	31.8 [29.7-34.0]	32.6 [25.7-39.4]	31.4 [28.9-33.8]	29.7 [25.2-34.2]	29.9 [24.1-35.8]	32.2 [29.3-35]	31.1 [28.9-33.3]	32.2 [22.9-41.6]
50-54	26.9 [26.5-27.3]	27.3 [25.2-29.5]	27.8 [21.3-34.4]	26.9 [24.5-29.3]	26.0 [21.9-30.1]	25.6 [20.1-31.0]	27.7 [24.9-30.4]	26.7 [24.6-28.8]	27.9 [19.0-36.7]
55-59	22.7 [22.3-23.0]	22.9 [20.8-24.9]	23.1 [16.9-29.4]	22.7 [20.4-24.9]	21.8 [18.0-25.7]	21.8 [17.0-26.6]	23.0 [20.4-25.7]	22.5 [20.4-24.5]	23.7 [15.3-32.0]
60-64	19.0 [18.7-19.3]	19.4 [17.7-21.1]	19.0 [13.5-24.5]	19.1 [17.1-21.0]	18.2 [15.0-21.3]	18.1 [14.2-22.0]	19.4 [17.2-21.6]	18.6 [16.9-20.3]	19.6 [12.5-26.6]
65-69	15.2 [14.9-15.6]	15.5 [13.9-17.0]	15.4 [10.7-20.1]	15.4 [13.7-17.1]	14.6 [11.8-17.4]	14.4 [11.0-17.7]	15.7 [13.7-17.7]	14.7 [13.2-16.3]	15.3 [8.8-21.9]
70-74	11.7 [11.4-12.0]	11.8 [10.4-13.3]	11.2 [6.7-15.7]	11.6 [10.0-13.1]	10.8 [8.3-13.4]	10.5 [7.4-13.7]	11.7 [9.9-13.6]	11.0 [9.5-12.5]	11.4 [5.2-17.6]
75-79	8.5 [8.2-8.9]	8.6 [7.2-9.9]	7.9 [3.7-12.1]	8.3 [6.9-9.7]	7.9 [5.5-10.2]	7.4 [4.6-10.3]	8.6 [6.8-10.3]	7.4 [5.9-8.9]	9.1 [3.9-14.3]
80-84	6.2 [5.9-6.5]	5.9 [4.6-7.3]	5.7 [1.9-9.5]	5.2 [3.9-6.6]	5.3 [3.0-7.7]	4.4 [1.6-7.2]	5.5 [3.7-7.3]	5.3 [3.7-7.0]	6.3 [1.3-11.3]
85-89	5.3 [5.0-5.6]	4.4 [3.3-5.5]	3.7 [0.0-7.4]	3.8 [2.6-5.1]	4.6 [2.5-6.7]	3.6 [0.0-7.3]	4.4 [3.1-5.7]	5.2 [3.5-6.9]	4.4 [0.1-8.7]
90+	4.1 [4.1-4.1]	3.1 [3.1-3.1]	5.0 [5.0-5.0]	2.7 [2.7-2.7]	3.0 [3.0-3.0]	5.3 [5.3-5.3]	2.2 [2.2-2.2]	4.7 [4.7-4.7]	2.3 [2.3-2.3]

Women

	Mainland	Korcula	Lastovo	Mljet	Hvar	Vis	Brač	Šolta	Pelješac
0-4	80.9 [80.5-81.3]	80.3 [77.8-82.8]	81.4 [73.8-89.0]	83.2 [75.1-91.3]	80.4 [77.4-83.3]	78.4 [72.7-84.1]	80.0 [77.0-82.9]	79.7 [73.8-85.7]	80.8 [77.3-84.3]
5-9	76.3 [75.9-76.7]	75.6 [73.4-77.9]	76.4 [68.8-84.0]	78.2 [70.1-86.3]	75.8 [73.3-78.2]	73.7 [68.5-78.9]	75.4 [72.9-78.0]	74.7 [68.8-80.7]	76.3 [73.4-79.3]
10-14	71.3 [70.9-71.7]	70.7 [68.5-72.9]	71.4 [63.8-79.0]	73.2 [65.1-81.3]	70.8 [68.3-73.2]	68.7 [63.5-73.9]	70.5 [68.0-73.0]	69.7 [63.8-75.7]	71.3 [68.4-74.3]
15-19	66.3 [65.9-66.7]	65.7 [63.5-67.9]	66.4 [58.8-74.0]	68.2 [60.1-76.3]	65.8 [63.3-68.2]	63.7 [58.5-68.9]	65.5 [63.0-68.0]	64.7 [58.8-70.7]	66.3 [63.4-69.3]
20-24	61.4 [61.0-61.8]	60.8 [58.6-63.0]	61.4 [53.8-69.0]	63.2 [55.1-71.3]	60.8 [58.3-63.2]	58.7 [53.5-63.9]	60.7 [58.2-63.1]	59.7 [53.8-65.7]	61.3 [58.4-64.3]
25-29	56.5 [56.1-56.9]	55.8 [53.6-58.0]	56.4 [48.8-64.0]	58.2 [50.1-66.3]	55.8 [53.3-58.2]	53.7 [48.5-58.9]	55.8 [53.4-58.1]	54.7 [48.8-60.7]	56.5 [53.6-59.3]
30-34	51.6 [51.2-51.9]	50.9 [48.8-53.0]	51.4 [43.8-59.0]	53.2 [45.1-61.3]	50.8 [48.4-53.3]	49.1 [44.3-53.9]	50.9 [48.6-53.2]	49.7 [43.8-55.7]	51.5 [48.6-54.3]
35-39	46.6 [46.3-47.0]	46.0 [43.9-48.1]	46.4 [38.8-54.0]	48.2 [40.1-56.3]	45.9 [43.5-48.3]	44.2 [39.6-48.9]	46.0 [43.7-48.3]	44.7 [38.8-50.7]	46.5 [43.7-49.3]
40-44	41.8 [41.4-42.1]	41.1 [39.0-43.1]	41.4 [33.8-49.0]	43.2 [35.1-51.3]	41.1 [38.8-43.4]	39.5 [35.1-43.9]	41.1 [38.9-43.4]	40.1 [34.8-45.4]	41.6 [38.8-44.4]
45-49	36.9 [36.6-37.3]	36.2 [34.2-38.2]	36.7 [29.4-44.0]	38.6 [31.0-46.2]	36.3 [34.1-38.5]	34.8 [30.5-39.1]	36.3 [34.2-38.5]	35.4 [30.5-40.3]	37.1 [34.5-39.6]
50-54	32.2 [31.9-32.6]	31.5 [29.5-33.4]	31.7 [24.4-39.0]	34.0 [26.9-41.0]	31.6 [29.4-33.7]	30.0 [25.8-34.2]	31.7 [29.6-33.8]	30.4 [25.5-35.3]	32.4 [30.0-34.8]
55-59	27.6 [27.3-28.0]	27.0 [25.1-28.9]	26.7 [19.4-34.0]	29.5 [23.4-35.5]	27.0 [25.0-29.1]	25.8 [21.8-29.7]	27.1 [25.1-29.1]	25.7 [21.2-30.3]	27.7 [25.5-30.0]
60-64	23.3 [23.0-23.6]	23.0 [21.5-24.5]	22.1 [15.3-28.8]	25.0 [19.9-30.2]	22.8 [21.0-24.6]	22.4 [19.7-25.1]	22.8 [21.1-24.6]	21.4 [17.7-25.0]	23.2 [21.2-25.2]
65-69	18.9 [18.6-19.2]	18.6 [17.2-20.0]	17.5 [11.1-23.9]	20.7 [15.9-25.4]	18.3 [16.6-19.9]	17.8 [15.4-20.3]	18.4 [16.8-20.1]	17.0 [13.7-20.3]	18.6 [16.6-20.5]
70-74	14.7 [14.4-15.0]	14.2 [12.9-15.5]	13.8 [8.7-18.9]	16.4 [12.3-20.5]	14.1 [12.6-15.6]	13.4 [11.1-15.7]	14.2 [12.7-15.8]	12.9 [10.1-15.8]	14.3 [12.5-16.2]
75-79	10.9 [10.7-11.2]	10.5 [9.4-11.7]	9.8 [5.1-14.6]	12.1 [8.2-15.9]	10.2 [8.8-11.5]	9.7 [7.6-11.8]	10.4 [9.0-11.9]	9.0 [6.7-11.3]	10.5 [8.8-12.2]
80-84	7.7 [7.4-8.0]	6.9 [5.8-8.0]	6.7 [2.6-10.8]	8.6 [5.0-12.2]	6.7 [5.4-8.0]	6.6 [4.6-8.6]	7.1 [5.6-8.5]	5.6 [3.5-7.6]	7.3 [5.6-8.9]
85-89	6.0 [5.7-6.2]	4.8 [4.0-5.7]	4.6 [2.0-7.3]	6.1 [3.2-9.0]	4.8 [3.8-5.8]	5.0 [3.4-6.6]	5.7 [4.6-6.8]	3.0 [1.7-4.3]	5.4 [4.0-6.9]
90+	4.4 [4.4-4.4]	3.2 [3.2-3.2]	1.4 [1.4-1.4]	3.9 [3.9-3.9]	2.9 [2.9-2.9]	3.5 [3.5-3.5]	4.2 [4.2-4.2]	1.5 [1.5-1.5]	4.6 [4.6-4.6]
